# Complete pathological response and negative postoperative ctDNA were not predictive of discontinuation of adjuvant crizotinib therapy in a patient with locally advanced MET ex14 skipping mutation-positive non-small cell lung cancer: a case report

**DOI:** 10.3389/fonc.2023.1164543

**Published:** 2023-07-24

**Authors:** Jiantao Wang, Wenqing Yao, Weiya Wang, Mingyu Fan, Kaili Huang, Zhenkun Liu, Daxing Zhu

**Affiliations:** ^1^Department of Thoracic Surgery, West China Hospital, Sichuan University, Chengdu, Sichuan, China; ^2^Lung Cancer Center, West China Hospital, Sichuan University, Chengdu, Sichuan, China; ^3^Department of Radiation Oncology, Cancer Center, West China Hospital, Sichuan University, Chengdu, Sichuan, China; ^4^Department of Pathology, West China Hospital, Sichuan University, Chengdu, Sichuan, China

**Keywords:** NSCLC, MET exon 14 skipping mutation, crizotinib rechallenge, neoadjuvant targeted therapy, case report

## Abstract

Neoadjuvant targeted therapy is an alternative treatment for locally advanced non-small cell lung cancer (NSCLC) patients with driver gene mutation. MET ex14 mutation is considered a driver gene, and crizotinib is the first oral tyrosine kinase inhibitor (TKI) for metastatic MET ex14 mutation-positive NSCLC patients. Here, we reported a case of a locally advanced NSCLC patient harboring MET ex14 mutation who achieved pathological complete response following neoadjuvant crizotinib therapy but developed rapid metastasis due to discontinuation of short-term postoperative adjuvant crizotinib therapy. Although no driver gene mutation was found via next-generation sequencing (NGS) with blood samples before discontinuation of adjuvant crizotinib, the patient was given crizotinib rechallenge. Fortunately, the patient achieved durable complete response. This suggested that neither pathological complete response nor negative circulating tumor DNA (ctDNA) could be an effective predictor for discontinuation of adjuvant targeted therapy. This case report demonstrated the potential of crizotinib as neoadjuvant therapy in MET ex14 mutation-positive NSCLC patients as well as the importance of long-term postoperative therapy even with negative ctDNA in blood.

## Introduction

Non-small cell lung cancer (NSCLC) patients harboring mesenchymal–epithelial transition factor exon 14 (MET ex14) skipping mutation accounts for less than 5% of all patients, and crizotinib has been reported as an effective treatment for advanced or metastatic lung adenocarcinoma patients with MET ex14 mutation (1). However, crizotinib as neoadjuvant therapy followed by postoperative adjuvant therapy in NSCLC patients with MET ex14 mutation has not been well defined. In addition, the efficacy of crizotinib rechallenge in the treatment of relapsed patients with MET ex14 mutation after successful adjuvant therapy followed by complete resection was not reported. Here, we report a case of successful crizotinib rechallenge in a locally advanced NSCLC patient harboring MET ex14 mutation who achieved pathological complete response following crizotinib neoadjuvant treatment but experienced rapid postoperative metastasis in the right upper lung due to discontinuation of short-term adjuvant crizotinib therapy, and rechallenge of crizotinib achieved durable radiological complete response.

## Case report

A 70-year-old male Chinese patient presented with a dry cough and was found to have an unresectable mass in diameter of 7.8 cm in his left upper lobe invading the left pulmonary artery trunk with multiple enlarged mediastinal lymph nodes by an enhanced chest CT. A diagnosis of lung adenocarcinoma was confirmed via bronchoscopy biopsy, and the patient was diagnosed as unresectable stage IIIB cT4N2M0 locally advanced lung adenocarcinoma after a comprehensive evaluation. Next-generation sequencing (NGS) was performed to detect if any driver gene mutation exists and targeted therapy could be a choice for this patient. Fortunately, MET ex14 mutation was found in both tumor tissues and blood samples by NGS, and the abundance of mutation was 12.2% for tissue samples and 0.2% for circulating tumor DNA (ctDNA) of blood samples. In addition, co-mutations of P53 p.C135F mutation of 7.0% abundance were detected only in tissue samples. Then, the patient was given crizotinib (250 mg, twice daily), which resulted in a significant decrease in the size and cavity of the primary lesion and was evaluated to have achieved radiological partial response after 2 months of treatment. Then, the patient underwent a pulmonary artery angioplasty and left upper lobe resection with systematic mediastinal lymph node dissection. For systematic mediastinal lymph node dissection, complete dissection with a total of 15 lymph nodes including mediastinal station 4L, station 5, station 6, station 7, station 9, station 10, and station 11 was performed. The pathology review of his specimen revealed non-viable tumor cells identified in the primary tumor lesion as well as in all resected lymph nodes, and only fibrosis and macrophage accumulation with necrosis were found, which were consistent with pathological complete response (pCR).

**Figure 1:**
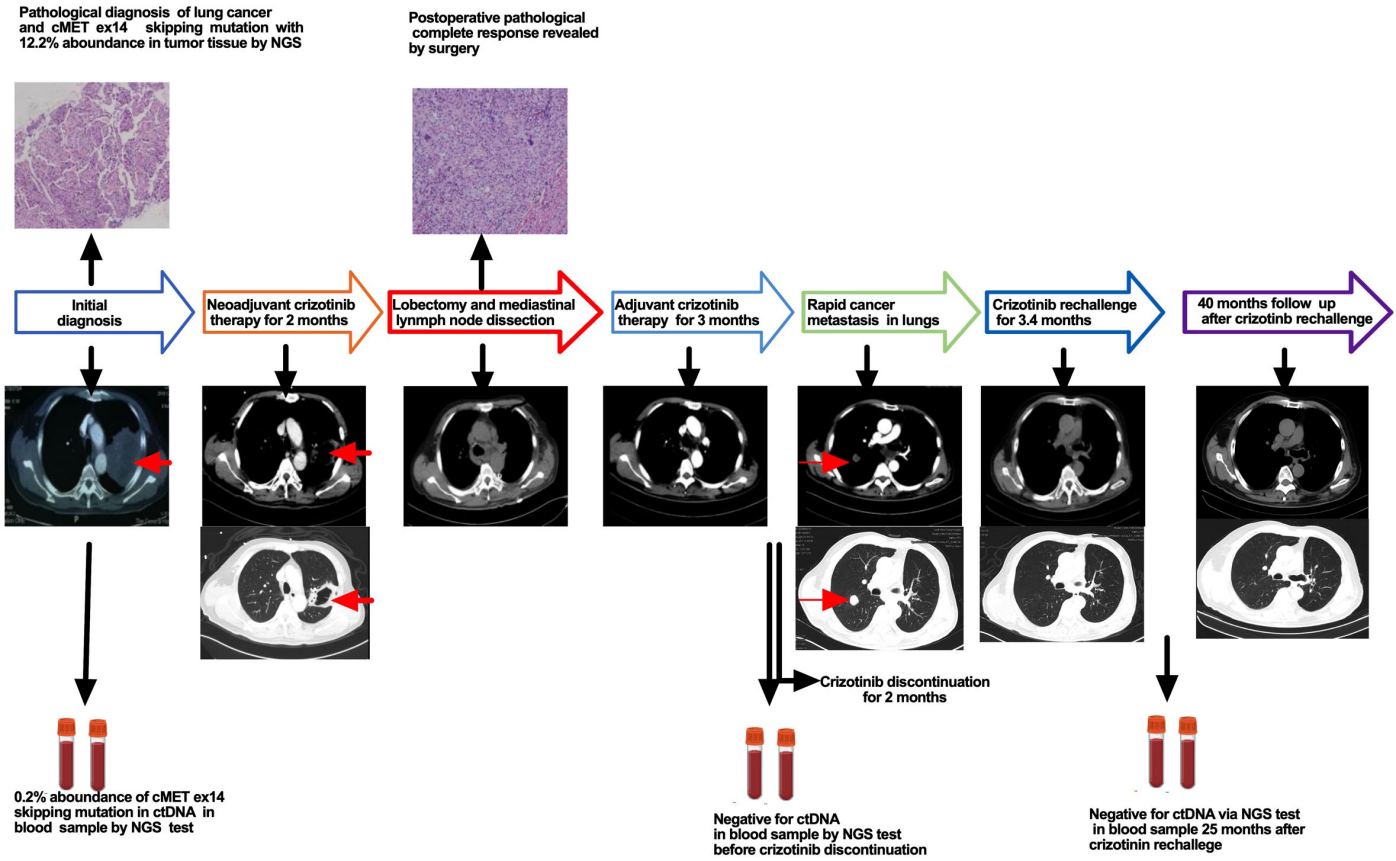
Clinical and treatment course of the patient. The red arrows indicate carcinoma.

The patient recovered well postoperatively and resumed crizotinib 3 weeks after surgery with good tolerance. Re-examination of next-generation sequencing of ctDNA by blood sample revealed no MET ex14 mutation 2 months after postoperative crizotinib adjuvant therapy. The patient stopped taking crizotinib 3 months after crizotinib adjuvant therapy due to personal issues. Unfortunately, rapid metastatic disease was found in the right upper lung 2 months after discontinuation of crizotinib. Considering short-term postoperative therapy with crizotinib to which the patient showed excellent response in neoadjuvant therapy, a rechallenge of crizotinib was given; as a result, a dramatic decrease in the tumor was found, and radiological complete response was achieved in 3.4 months after crizotinib rechallenge. The patient underwent crizotinib therapy for more than 3 years after achieving radiological complete response (CR) with good tolerance. The patient received another NGS test via blood sample after 25 months of crizotinib rechallenge, and no MET ex14 mutation was found. Until now, the patient has received follow-up care and has disease-free survival for nearly 40 months (the treatment process is illustrated in **Figure 1**).

## Discussion

Neoadjuvant chemotherapy has been proven of limited efficacy in locally advanced NSCLC patients; however, unlike conventional chemotherapy, the role of targeted therapy as neoadjuvant therapy was not well defined in these patients (2). Recently, CTONG1103 has proved that neoadjuvant erlotinib therapy achieved better progression-free survival (PFS) when compared to chemotherapy in NSCLC patients with EGFR mutation (3). Also, in other uncommon driver genes mutation-positive locally advanced NSCLC patients, several studies with small samples revealed that neoadjuvant targeted therapy followed by surgery and adjuvant targeted therapy might be an alternative (4).

MET ex14 mutation is considered a driver gene in NSCLC, and crizotinib is the first oral tyrosine kinase inhibitor (TKI) for the treatment of metastatic NSCLC patients with MET ex14 mutation (5). In 2020, capmatinib, a new MET ex14 mutation-targeting TKI, was approved by the Food and Drug Administration (FDA) and recommended as a preferred choice for metastatic NSCLC patients with MET ex14 mutation (6). Recently, there is a clinical trial named “GEOMETRY-N trial”, which aimed to evaluate the efficacy and safety of neoadjuvant and adjuvant capmatinib therapy in improving clinical outcomes in patients with MET ex14 mutation or high-level MET amplification NSCLC patients, and capmatinib was designed for use as adjuvant therapy for up to 3 years. Recently, Rotow reported a case of pCR due to neoadjuvant crizotinib in a MET ex14 mutation locally advanced NSCLC patient whose progression-free survival was not established at the end of a 6-month-long follow-up because of persistent postoperative adjuvant crizotinib therapy (7). In the current case, the initial diagnosis was in 2018, only crizotinib was available, and the patient achieved pCR due to neoadjuvant crizotinib therapy. Furthermore, no MET ex14 mutation was found by ctDNA test via blood sample after 2 months of postoperative crizotinib adjuvant therapy. However, the patient experienced rapid metastasis after discontinuation of short-term postoperative adjuvant crizotinib therapy. Zhong reported an ALK fusion mutation NSCLC patient who achieved pCR but developed rapid relapse due to discontinuation of crizotinib adjuvant therapy with negative ctDNA following 3.6 months of crizotinib adjuvant therapy (8). Altogether, these data indicated that neither complete pathological response nor negative ctDNA could be an effective predictor for discontinuation of short-term adjuvant targeted therapy in locally advanced NSCLC patients harboring MET ex14 mutation with complete surgical resection and also indicated the importance of long-term continuous postoperative crizotinib therapy even with negative ctDNA. To the best of our knowledge, this is the first case of discontinuation of adjuvant therapy that led to rapid metastasis in a patient with MET ex14 mutation who achieved pCR following neoadjuvant crizotinib targeted therapy, and rechallenge of crizotinib achieved complete response quickly and resulted in long-time disease-free survival. In conclusion, neoadjuvant targeted therapy with crizotinib could be a choice for locally advanced NSCLC patients harboring MET ex14 mutation, and long-time continuous adjuvant therapy is important even in patients who achieved pCR but have no driver gene mutation in ctDNA via NGS with blood samples.

## Data availability statement

The datasets presented in this article are not readily available because of ethical/privacy restrictions. Requests to access the datasets should be directed to the corresponding author.

## Ethics statement

The studies involving human participants were reviewed and approved by Ethics Committee of West China Hospital, Sichuan University. The patients/participants provided their written informed consent to participate in this study. Written informed consent was obtained from the individual(s) for the publication of any potentially identifiable images or data included in this article.

## Author contributions

JW: Investigation, data organization, writing – original draft. WY: Data collection. WW: Data collection. MF: Writing - review and editing. KH: Writing - review and editing. ZL: Writing - review and editing. DZ: Supervision, writing - review and editing.
